# Targeting HER2^+^ breast cancer: the TBK1/IKKε axis

**DOI:** 10.18632/oncoscience.18

**Published:** 2014-03-06

**Authors:** Zhe Jiang, Jeff C. Liu, Philip E.D. Chung, Sean E. Egan, Eldad Zacksenhaus

**Affiliations:** ^1^ Division of Advanced Diagnostics, Toronto General Research Institute - University Health Network, Toronto, Ontario, Canada; ^2^ Program in Developmental and Stem Cell Biology, The Hospital for Sick Children, Department of Molecular Genetics, University of Toronto

**Keywords:** Breast cancer, HER2, therapy, TBK1, IKKε

## Abstract

HER2^+^ breast cancer (BC) is a highly aggressive subtype, affecting ~20% of BC patients. Current treatments include adjuvant or neoadjuvant chemotherapy plus anti-HER2 agents such as trastuzumab, a monoclonal antibody directed against HER2. Despite improvement in disease free survival, most patients eventually succumb to metastatic disease, which is largely incurable. Consequently, there is an urgent need to identify novel drugs that can efficiently kill HER2^+^ BC and/or potentiate the effect of existing anti-HER2 therapies. We performed a lenti-viral shRNA kinome screen on non-adherent mouse Her2/Neu tumorspheres and identified TBK1, a non-canonical IκB kinase (IKK), as the most potent target [1]. TBK1 knock-down, or treatment with TBK1-II, a drug that efficiently inhibits TBK1 and its close relative IKKε (IKBKE), suppressed growth of human HER2^+^ BC cells and induced cellular senescence. Senescence was associated with inhibition of phosphorylated/active p65-NFkB and induction of the cell cycle inhibitor, p16^ink4a^. In addition, TBK1-II cooperated with lapatinib, a EGFR/HER2 inhibitor, to accelerate apoptosis *in vitro* and suppress tumor growth in a xenograft model of HER2^+^ BC. Thus, TBK1/IKKε inhibitors may improve treatment of HER2^+^ BC in cooperation with anti-HER2 therapy.

HER2^+^ breast cancer (BC) is a highly aggressive subtype, affecting ~20% of BC patients. Current treatments include adjuvant or neoadjuvant chemotherapy plus anti-HER2 agents such as trastuzumab, a monoclonal antibody directed against HER2. Despite improvement in disease free survival, most patients eventually succumb to metastatic disease, which is largely incurable. Consequently, there is an urgent need to identify novel drugs that can efficiently kill HER2^+^ BC and/or potentiate the effect of existing anti-HER2 therapies. We performed a lenti-viral shRNA kinome screen on non-adherent mouse Her2/Neu tumorspheres and identified TBK1, a non-canonical IκB kinase (IKK), as the most potent target [1]. TBK1 knock-down, or treatment with TBK1-II, a drug that efficiently inhibits TBK1 and its close relative IKKε (IKBKE), suppressed growth of human HER2^+^ BC cells and induced cellular senescence. Senescence was associated with inhibition of phosphorylated/active p65-NF-κB and induction of the cell cycle inhibitor, p16^ink4a^. In addition, TBK1-II cooperated with lapatinib, a EGFR/HER2 inhibitor, to accelerate apoptosis *in vitro* and suppress tumor growth in a xenograft model of HER2^+^ BC. Thus, TBK1/IKKε inhibitors may improve treatment of HER2^+^ BC in cooperation with anti-HER2 therapy.

Many cancers including HER2^+^ BCs are organized in a hierarchy with tumor initiating cells (TICs) at its apex, and non-TICs, which descend from TICs but have lost tumorigenic potential as the tumor bulk. We previously identified TICs from MMTV-Her2/Neu tumor cells; by multiple criteria, cells that gave rise to non-adherent tumorspheres in ultra-low attachment plates were indistinguishable from TICs [2, 3]. We have now shown that the non-adherent Her2/Neu tumorspheres retain high TIC frequency, whereas adherent monolayer cells quickly lose the TIC fraction [1]. We also found that after three passages, both adherent and non-adherent cultures become enriched for cells with spontaneous p53-mutations that occur in about half of primary MMTV-Her2/Neu tumors. These p53-mutant/Her2^+^ mouse mammary tumor cells better resemble human HER2^+^ BC where p53 is mutated in ~70% of cases.

To identify new targets for HER2^+^ BC, we performed a lenti-virus shRNA kinome screen on the adherent and non-adherent p53-mutant/Her2^+^ tumor cells using ~5 independent shRNAs per gene for 520 individual kinases. shRNAs that preferentially suppressed sphere growth, or both sphere and monolayer, but not monolayer growth only were studied further. We also selected genes for which at least two independent shRNAs (of the 5 tested) scored positive, and that their knockdown did not suppress growth of immortalized mammary epithelial cells by more than 25%. After validation, essential genes for growth of sphere or sphere plus monolayer cells included those on the MAP kinase pathway (A-Raf, Raf1, mapkapk3) and TGFβ-receptor superfamily, which were previously linked to HER2^+^ BC. In addition, we identified Camk2d, Cask and Stk25 as well as the pro-autophagy related factor/unc-51-like kinase (Atg1/Ulk1). Consistent with the latter, we showed that mouse and human HER2^+^ BC cells are highly sensitive to autophagy inhibitors. Finally, our screen identified Map3k14 (NF-κB inducing kinase - NIK) and the non-canonical IκB Kinase (IKK) TANK-binding kinase 1 (TBK1). Map3k14/NIK and TBK1 induce NF-κB through different signaling pathways [4]. Their identification in our screen suggests that NF-κB activation is essential for growth or survival of HER2- driven BC cells.

TBK1 and the related kinase IKKε (IKBKE) play important roles in innate immunity by regulating interferon and NF-κB signaling [4, 5]. In addition, TBK1 was identified in a synthetic lethal screen for genes that could kill lung cancer cells driven by activated K-RAS. IKKε is amplified and overexpressed in ~30% of BC and can transform breast epithelial cells through activation of NF-κB [4]. We found that shRNA-mediated knockdown of TBK1 efficiently suppressed growth of both mouse and human HER2^+^ BC cells. Moreover, TBK1-II, a specific inhibitor for both TBK1 and IKKε, dramatically suppressed growth of HER2^+^ BC cells. As expression of TBK1 and IKKε varies in different BC cells, their contribution to TBK1-II inhibition is expected to vary accordingly. Importantly, TBK1-II cooperated with lapatinib to kill HER2^+^ BC cells in xenograft assays in vivo. Although TBK1-II is potent and specific, our pharmacokinetic analysis revealed that it has a relatively short half-life in vivo. Thus, an immediate objective is to identify a selective, potent and stable TBK1/IKKε inhibitor.

Several reports have indicated that TBK1 maintains cell survival by directly phosphorylating and activating AKT/PKB, or by inducing BCL-xL through NF-κB [4, 6]. Surprisingly, in HER2^+^ BC cells, we found that genetic or pharmacological inhibition of TBK1, or TBK1 plus IKKε, did not induce apoptotic cell death or necrosis. Accordingly, TBK1/IKKε-inhibition did not affect AKT phosphorylation or BCL-xL expression. Instead, inhibition of these non-canonical IKKs led to enlarged cells with >8N DNA content, and suppression of p65-NF-κB (RelA) phosphorylation on serine 536, ultimately inducing cellular senescence.

NF-κB proteins exert pro- or anti-senescence effects in different contexts. In HER2^+^ BC, we showed that TBK1/IKKε maintains p65-NF-κB in an active state, thereby putting the brakes on cellular senescence (Fig. 1). We also observed a dramatic increase in expression of p16^ink4a^, a CDK4/6-inhibitor that prevents phosphorylation/ inactivation of the tumor suppressor pRb and its relatives, p107 and p130. The epistatic interaction between TBK1/IKKε, p16^ink4a^ and NF-κB is yet to be established. The most parsimonious model is that phosphorylated/active p65-NFκB actively suppresses the p16^ink4a^ promoter, and that this suppression is relieved in response to TBK1/IKKε-inhibition and p65-NF-κB dephosphorylation.

**Fig 1:**
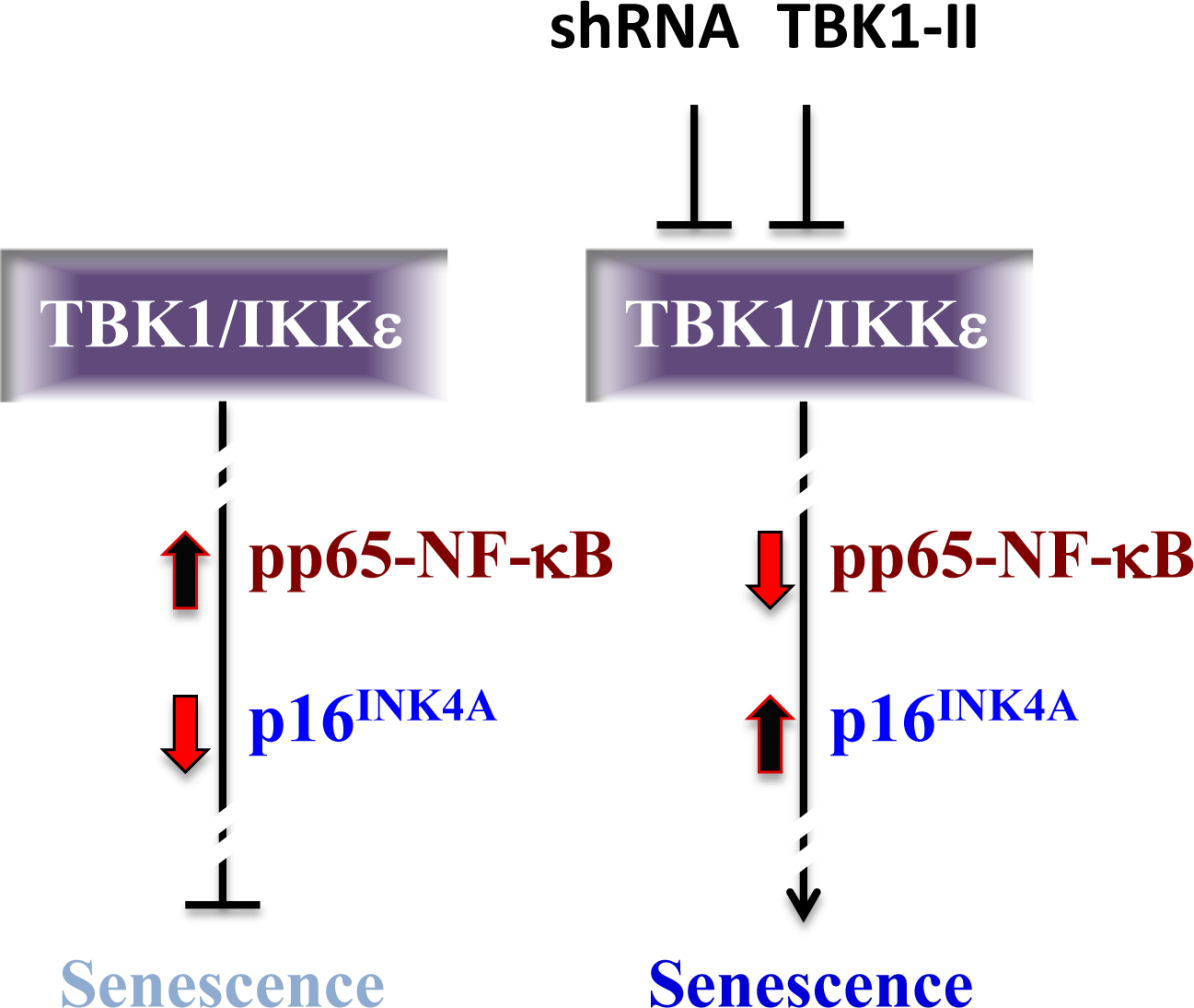
A model for TBK1/IKKε-dependent inhibition of cellular senescence in HER2+ BC cells The TBK/ IKKε axis maintains p65-NF-κB active/phosphorylated thereby inhibiting cellular senescence. Knock-down of TBK1 or pharmacological inhibition TBK/IKKε blocks p65-NF-κB phosphorylation and induces p16ink4a expression, causing cell senescence.

Importantly, TBK1-II plus lapatinib treatment did not increase cell senescence but instead accelerated apoptotic cell death relative to lapatinib treatment alone (Fig. 2). Thus, in the context of combination therapy, inhibition of TBK1/IKKε may potentiate the effect of anti-HER2 inhibitors and improve clinical outcome. TBK1 has recently been shown to control the response of ER^+^ BC cells to anti-estrogen therapy [7, 8], and to ameliorate experimentally-induced obesity in mice [9]. These other applications may help speed up the development of TBK1/ IKKε therapeutics and their use for treatment of HER2^+^ BC.

**Fig 2:**
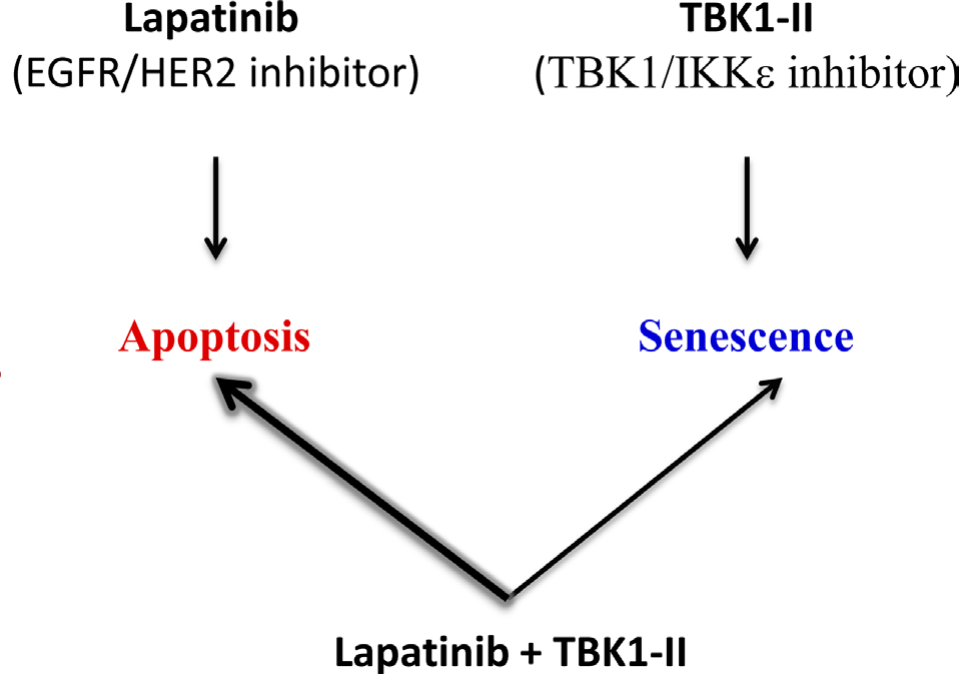
A TBK1/IKKε inhibitor (TBK1-II) cooperates with the EGFR/HER2 inhibitor (lapatinib) to further promote apoptotic cell death, but not cellular senescence
